# N-oleoylethanolamine − phosphatidylcholine complex loaded, DSPE-PEG integrated liposomes for efficient stroke

**DOI:** 10.1080/10717544.2021.2008058

**Published:** 2021-11-29

**Authors:** Xiangrui Yang, Shichao Wu

**Affiliations:** a Department of Nuclear Medicine (PET Center), Xiangya Hospital, Central South University, Changsha, PR China; b Key Laboratory of Nanobiological Technology of National Health Commission, Xiangya Hospital, Central South University, Changsha, PR China; c National Clinical Research Center for Geriatric Disorders, Xiangya Hospital, Central South University, Changsha, PR China

**Keywords:** Stroke, N-oleoylethanolamine (OEA), neuroprotective, drug delivery, DSPE-PEG

## Abstract

Causing more and more deaths, stroke has been a leading cause of death worldwide. However, success in clinical stroke trials has remained elusive. N-oleoylethanolamine (OEA) was an endogenous highly hydrophobic molecule with outstanding neuroprotective effect. In this article, hydrogen bonds were successfully formed between OEA and soybean phosphatidylcholine (SPC). The synthetic OEA-SPC complex and DSPE-PEG were self-assembled into liposomes (OEA NPs), with OEA-SPC loaded in the core and PEG formed a hydrophilic shell. Hence, highly hydrophobic OEA was loaded into liposomes as amorphous state with a drug loading of 8.21 ± 0.18 wt%. With fairly uniform size and well-distributed character, the OEA NPs were systemically assessed as an intravenous formulation for stroke therapy. The results indicated that the administration of OEA NPs could significantly improve the survival rate and the Garcia score of the MCAO rats compared with free OEA. The TTC-stained brain slices declared that the cerebral infarct volume and the edema degree induced by MCAO could be decreased to an extremely low level *via* the administration of OEA NPs. The Morris water maze (MWM) test suggested that the spatial learning and memory of the MCAO rats could also be ameliorated by OEA NPs. The immunofluorescence assay stated that the apoptosis of the neurons and the inflammation within the brain were greatly inhibited. The results suggest that the OEA NPs have a great chance to develop OEA as a potential anti-stroke formulation for clinic application.

## Introduction

Responsible for approximately 11% of total deaths in 2019, stroke has been a leading cause of death worldwide (Virani et al., 2020). However, success in clinical stroke trials has remained elusive, although an overstated efficacy might have been obtained in animal models (Gonzalez-Nieto et al., 2020). The substantial difference in brain structure and coagulation system between rodents and humans might be the most likely cause of the translation (Howells et al., 2010; Savitz et al., 2017). In addition to the complex pathophysiology of stroke, different pathophysiological processes have different relative contributions to human stroke than in animal models (Sommer, 2017; Ballarin & Tymianski, 2018; Chen et al., 2018; Lee et al., 2018). Moreover, human stroke might involve additional processes that could not be present in animal models.

Many patients with acute ischemic stroke firstly received thrombolytic therapy to open the occluded blood vessel (Powers et al., 2018; Whelton et al., 2018). Nevertheless, large vessel reperfusion would induce reactive oxygen species (ROS) and inflammation, which might damage the neurons and lead to disability (Gilgun-Sherki et al., 2002; Valko et al., 2007; Forrester et al., 2018). By clearing the ROS, edaravone was proven as an effectively neuroprotective compound (Zhang et al., 2005; Yoshida et al., 2006). Although several critical problems still remained, such as the inefficiency in crossing the blood–brain barrier (BBB), the overhigh dosage, and the severe side effects (Bao et al., 2018), edaravone has been the commonly-used drug in the clinic. To a certain extent, the reduction of ROS might help to weaken the inflammation. However, this is far from effectively relieving the inflammation and its damage to the brain, since the reduced CBF, BBB dysfunction, and excess calcium can also induce severe inflammation (Campbell et al., 2019). And the inflammation would cause protein misfolding, cytoskeletal breakdown, and the increase of ROS, which would ultimately induce apoptotic neuron death (Dirnagl et al., 1999; Shi et al., 2019). Hence, modulating the inflammatory process plays a paramount role in stroke-fighting (Nakamura and Shichita, 2019; Peng et al., 2019; Stoll & Nieswandt, 2019; Rawlinson et al., 2020). Nevertheless, it’s still a challenging clinical problem to comb out or even reduce the inflammation in stroke. Actually, only limited data are available regarding the inflammatory response in humans (Wimmer et al., 2018).

As the therapeutic potential of manipulating the inflammatory response to stroke has not achieved progress for a long time, our team confirmed that OEA possessed excellent neuroprotective effect mainly *via* alleviating the inflammation (Zhou et al., 2012; Yang et al., 2015; Zhou et al., 2017). OEA could protect mice from acute cerebral ischemic injury by enhancing PPARα signaling, and chronic OEA administration reversed spatial cognitive impairments and even promoted neurogenesis and neuroplasticity in rats after focal cerebral ischemia (Yang et al., 2016; Zhao et al., 2018; Luo et al., 2019). What’s more, the distinctive superiority of OEA might be endogenous, which would help to address some bottlenecks in clinical trials. However, the extreme insolubility of OEA has become a major obstacle to its application. Plus the fact that OEA could be enzymatically hydrolyzed and rapidly deactivated, OEA could not be used in the clinic in its current form (Wulff-Perez et al., 2014). To save the OEA, we turned to the nanoparticles drug delivery system (NDDS). We prepared the orally administrated OEA NDDS as an efficient anti-stroke formulation, which could greatly improve the solubility and the bioavailability of OEA (Yang et al., 2019). The OEA NDDS could efficiently reduce the cerebral infarct volume, the edema degree, and the inflammation in the MCAO rats. Although the OEA NDDS possessed an excellent neuroprotective effect, the onset time of the oral pharmaceutical formulation might be too long for acute stroke. In other words, the oral OEA NDDS might be more suitable for stroke convalescence.

Hence, in this work, we integrated OEA into liposomes with PEG chains on the surface for stroke therapy. *Via* forming hydrogen bonds between OEA and SPC, the drug was efficiently and firmly loaded into OEA NPs. With PEG on the surface, the OEA NPs possessed a small and uniform size of about 100 nm. And the OEA NPs were well-distributed and stable enough for intravenous administration. Then systemic *in vivo* experiments were performed to assess the neuroprotective effect of the OEA NPs.

The objective of this work was to formulate OEA into liposomes that aim at enhancing its bioavailability and increasing its neuroprotective effect for clinical intravenous use. We expected that these hydrogen-bond-based, OEA-loaded liposomes might be a useful formulation for stroke therapy.

## Material and methods

### Materials

All chemical reagents were of analytical grade and used without further purification unless otherwise stated. OEA (purity grade > 90.0%), SPC (purity grade >90%) were obtained from Shanghai Macklin Biochemical Co., Ltd (Shanghai, China). DSPE-PEG (MWCO 2000) was purchased from Ponsure Biotechnology Co., Ltd (Shanghai, China). Dichloromethane (DCM), and tetrahydrofuran (THF) were provided by Sinopharm Chemical Reagent Co., Ltd. (Shanghai, China). 2, 3, 5-triphenyltetrazolium chloride (TTC) were obtained from Shanghai yuanye Bio-Technology Co., Ltd (Shanghai, China).

### Preparation of OEA − SPC complex (OEA-SPC)

OEA (3 mg) and SPC (12 mg) were codissolved in a glass pressure vessel with THF (8 mL), accompanied with vigorous agitation at 40 °C for 8 h. Then, THF was removed by vacuum rotary evaporation (RE-5299; YUHUA, Gongyi, China).

### Preparation of OEA-SPC & DSPE-PEG nanoparticles (OEA NPs)

The OEA NPs were synthesized *via* a nanoprecipitation technique. Briefly, OEA-SPC (3 mg of OEA) was dissolved in DCM, which was then dropwise (2 mL/min) introduced into distilled water (40 mL) containing DSPE-PEG under sonication. And the system turned into a stable, white O/W suspension. Subsequently, DCM was evaporated from the emulsion by magnetic stirring overnight, producing a clear suspension and resulting in the formation of the OEA NPs. Lastly, the suspensions were filtered through a 0.22 μm polycarbonate membrane. Then, the suspension was centrifuged at 5000 rpm and lyophilized for 24 h to obtain the dry powder.

### Characterization

The OEA − SPC complex was analyzed with H^1^NMR (AVANCE III 400 MHz). Free OEA and SPC were used as control. Morphology of the OEA NPs was examined by TEM (Tecnai G2 F20, FEI, USA) at 200 kV. The Size and zeta-potential were determined by Malvern Zetasizer Nano-ZS (Malvern Instruments, Malvern, UK). Three parallel measurements were performed to determine the average values. The drug loading content of OEA in OEA NPs was determined by liquid chromatography-mass spectrometry (LC-LTQ-Orbitrap, Thermo Fisher, USA). The drug loading and encapsulation efficiency was calculated by Equations (1) and (2), respectively.

(1) $$\text{Drug loading content of OEA}(\%)=\frac{\left(\text{weight of OEA in NPs}\right)}{\left(\text{weight of NPs}\right)}\times 100\%$$

(2) $$\text{Entrapment efficiency of OEA}(\%)=\frac{\left(\text{weight of OEA in NPs}\right)}{\left(\text{weight of feeding OEA}\right)}\times 100\%$$

### In vitro drug release study

The *in vitro* drug release studies of OEA NPs were performed *via* the dialysis technique. Free OEA, OEA-SPC NPs, and OEA NPs were dispersed in PBS buffer solution (8 mL) and placed in dialysis bags (MWCO 3500 Da). Then, the bags were immersed in PBS (0.1 mol/L, 200 mL, pH 7.4) and continuously oscillated in a shaker incubator (200 rpm, 37 °C). All samples were assayed by LC-MS.

### Drug administration

The OEA NPs (0.35 mg/mL) were dissolved in saline under ultrasonic breaking. OEA (0.35 mg/mL) and Twain’s 80 were codissolved in saline under strong shaking. Drugs (1.5 mg/kg, iv) were administered with reperfusion, and daily for 14 consecutive days after ischemia.

### Preparation of the focal cerebral ischemia model

Focal cerebral ischemia was induced by middle cerebral artery occlusion (MCAO) in adult male Sprague–Dawley (SD) rats (250 g), as previously described. Briefly, rats were anesthetized with chloral hydrate (380 mg/kg, ip). The 4-0 silicon rubber-coated nylon monofilament was inserted into the right internal carotid artery (ICA) through the external carotid stump, and past the ECA/ICA bifurcation to occlude the origin of the middle cerebral artery (MCA) at the junction of the circle of Willis. The monofilament was kept for 1.5 h and then withdrawn. Sham-operated rats were treated with an identical surgery without inserting the intraluminal filament. Rats would be excluded if the hemorrhage was found in the brain slices or at the base of the circle of Willis during postmortem examination.

### The TTC-stained slices

The MCAO rats (*n* = 6) were decapitated at 24 h post-reperfusion, and the brains were removed rapidly and cut into five 2.5-mm thick coronal sections, which were then stained with standard TTC (2%, 10 min, 37 °C) followed by overnight immersion in formalin (10%). Images of the stained brain slices were captured with a digital camera. The infarct area of the slices was measured with Image Tool version 2.0 software (UTHSCSA, San Antonio, Tex., USA) and calculated as the infarct area thickness (2.5 mm). The summed infarct volume of all brain slices was calculated as the total infarct volume. To determine the extent of ipsilateral edema degree, the increased percentage in the ischemic hemisphere volume was calculated by Equation (3):

(3) $$\text{Ipsilateral edema degree}(\%)=\frac{\left(\text{ipsilateral volume}-\text{contralateral volume}\right)}{\left(\text{contralateral volume}\right)}\times 100\%$$

### Measurement of the survival rate

The survival rate of the rats was recorded. (*n* = 12 rats for the sham group and the nanodrug group, and *n* = 24 rats for the MCAO group and the OEA group). And the rats were involved in the following experiments.

### Evaluation of neurological deficit

At 1, 3, 5, 7, and 14 d after reperfusion, rats (*n* = 10) were evaluated neurologically by a single examiner who was blinded to the animal groups using the Garcia score. Animals were given a score of 0–18 (higher scores indicate greater function).

### Morris water maze task

Morris water maze (MWM) task was carried out between day 15 and 20 post-reperfusion. The protocol followed our previous report. Acquisition training consisted of 5-d conditioning with 5 trials per day from day 15 to 19. For each trial, the rats (*n* = 10 for each group) were placed in water facing the wall of the maze and allowed to swim for a maximum of 120 s. If the rats found the platform, they were allowed to stay on it for 15 s. If they cannot find the platform within 120 s, they would be guided to the platform and remain on it for 15 s. The escape latency was recorded. On day 20, the platform was removed and the rats were given one 120-s retention probe test. The swimming traces of the rats were recorded by a video camera with a computer *via* an image analyzer. The number of times each animal crossed the position where the platform had been previously located was recorded by the analyzer.

### Immunofluorescence staining and cell counting

Rats (21 d after reperfusion) were anesthetized with chloral hydrate and perfused transcardially with ice-cold saline and subsequently paraformaldehyde (4%). The brains were extracted and dehydrated with gradient sucrose (10, 20, and 30%) and then coronal sectioned (30 μm) with a vibrating microtome (Leica, Wetzlar, Germany). The sections were incubated in PBS (0.5% Triton X-100 and 10% goat serum) for 60 min at room temperature, following by incubation with rabbit polyclonal, TUNEL (Elabscience, CHN), rabbit polyclonal Iba-1 (Abcam, Cambridge, UK), or GFAP (CST) at 4 °C overnight. After three PBS rinses, sections were incubated with Alexa Fluor 594 donkey anti-rabbit IgG (Abcam, Cambridge, UK), or Alexa Fluor 488 donkey anti-rabbit IgG (Abcam, Cambridge, UK). The number of TUNEL, GFAP, or Iba1 positive cells was observed by fluorescence confocal microscopy (EX61, Olympus, Tokyo, Japan). The positive cells were counted in three randomly chosen squares of identical size (460 × 460 μm).

### Statistical analysis

The statistical significance of treatment outcomes was assessed using one-way/two-way analysis of variance for the differences within treatments followed by Tukey’s post hoc test (Prism version 7 for Windows, GraphPad Software Inc., La Jolla, CA); *p* < .05 was considered statistically significant in all analyses (95% confidence level).

## Results and discussion

### Preparation of OEA-SPC&DSPE-PEG nanoparticles (OEA NPs)

**Figure 1.**
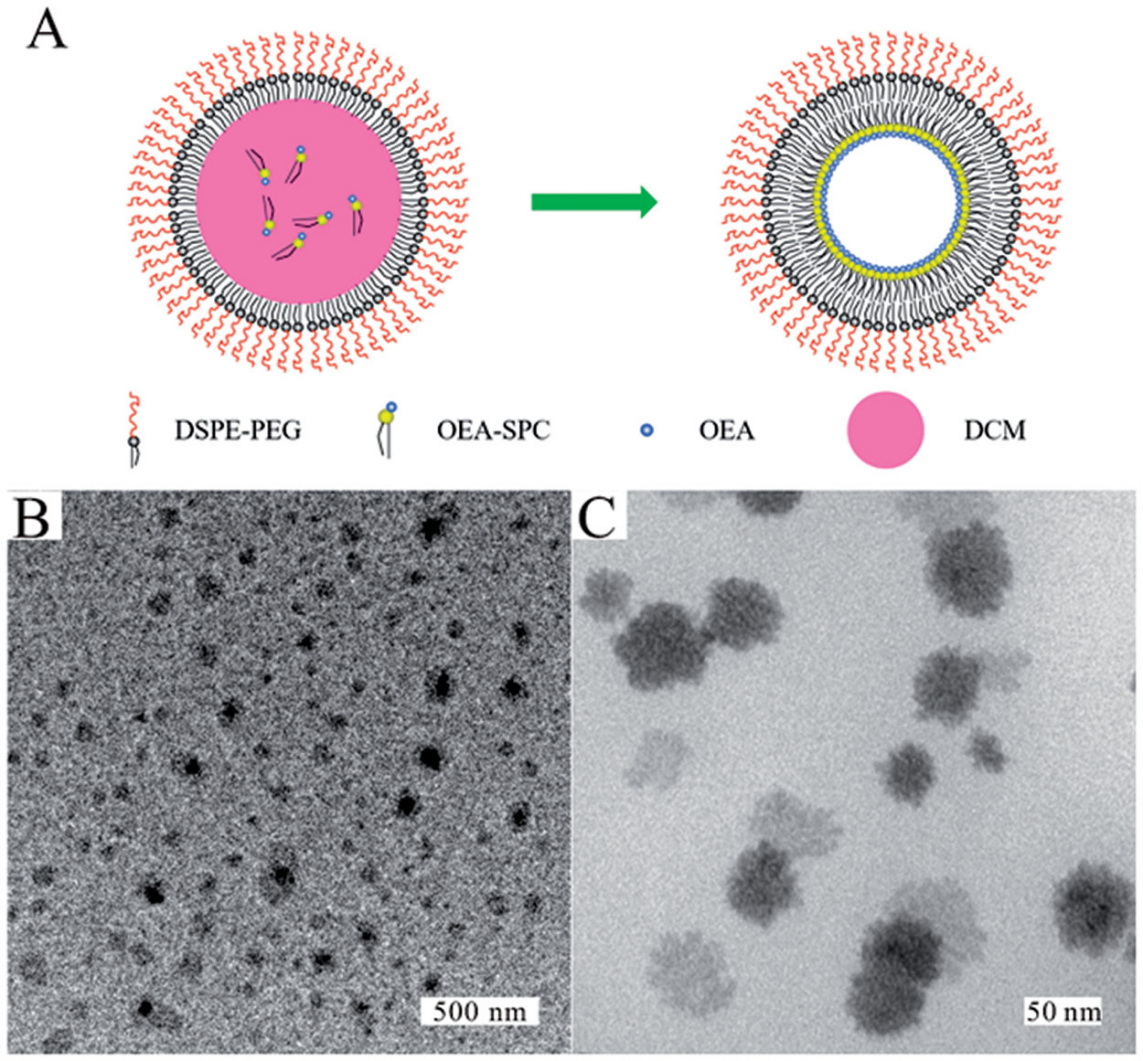
(A) Illustration of the preparation process of the OEA NPs. (B) The TEM images of the OEA NPs.

First, OEA was integrated by forming hydrogen bonds with SPC at a 1:4 weight ratio according to our previous study (Yang et al., 2019). The formation of the hydrogen bonds was confirmed by H^1^NMR analysis. As shown in Figure S1, the peaks of –NH– (7.76 ppm) and –OH (4.67 ppm) in the spectra of OEA, and the peak of phosphatidylcholine (3.45 ppm) in the spectra of SPC appeared to obviously weaken and slightly shift in the spectra of the OEA–SPC complex. The result illustrated the formation of hydrogen bonds between OEA and SPC, which was in accordance with the previous studies (Yang et al., 2019). Then, the OEA NPs were prepared *via* ultrasonic emulsification technology. Briefly, the OEA-SPC complex was dissolved in DCM, which was then dropped slowly into DI water containing DSPE-PEG under ultrasonic conditions. With the dispersion of DCM droplets in water, the system turned into a stable, white O/W suspension. Owing to the amphiphilic property of the DSPE-PEG molecules, the hydrophobic groups (DSPE) were arranged around the organic droplets and the hydrophilic PEG stretched into the continuous phase. When stirring overnight, DCM was volatilized, leading to the arrangement of the OEA-SPC complex inside the droplets and forming liposomes (Figure 1(A)).

Since the OEA NPs were prepared for intravenous administration, their properties, such as the size and the distribution were strictly required. And we performed an orthogonal experiment to optimize the preparation method. The experiment conditions and the results are shown in Tables S1 and S2, respectively. It could be found that the size decreased with the ratio of OEA-SPC to DSPE-PEG. However, when the ratio was more than 1.75:1, a thick polymer layer could appear under the transmission electron microscope (Figure S2), illustrating the excess of DSPE-PEG. When the ratio was less than 1:1, the size of the liposomes might be too large for intravenous administration. The ultrasonic power was also a paramount factor influencing the property of the liposomes. High power would induce imperfect liposomes and low power led to large liposomes. The concentration of the DSPE-PEG also affected the property of the liposomes. High concentration could reduce the size but increase the PDI of the liposomes. Taken all these factors into account, the ratio of 1:1.3, ultrasonic power of 200 W, and concentration of 0.5 mg/mL were chosen as the optimized preparation conditions. The optimized OEA NPs possessed a drug loading of 8.21 ± 0.18 wt%, an encapsulation efficiency of 93.6 ± 2.5%, a size of 76.3 ± 5.6 nm (Figure S3), a zeta potential of 15.4 ± 3.1 mV, and a PDI of 0.104 ± 0.021. These data indicated that the OEA NPs exhibited a fairly uniform size and well-distributed character, which could be visualized in TEM images (Figure 1(B,C)).

**Figure 2.**
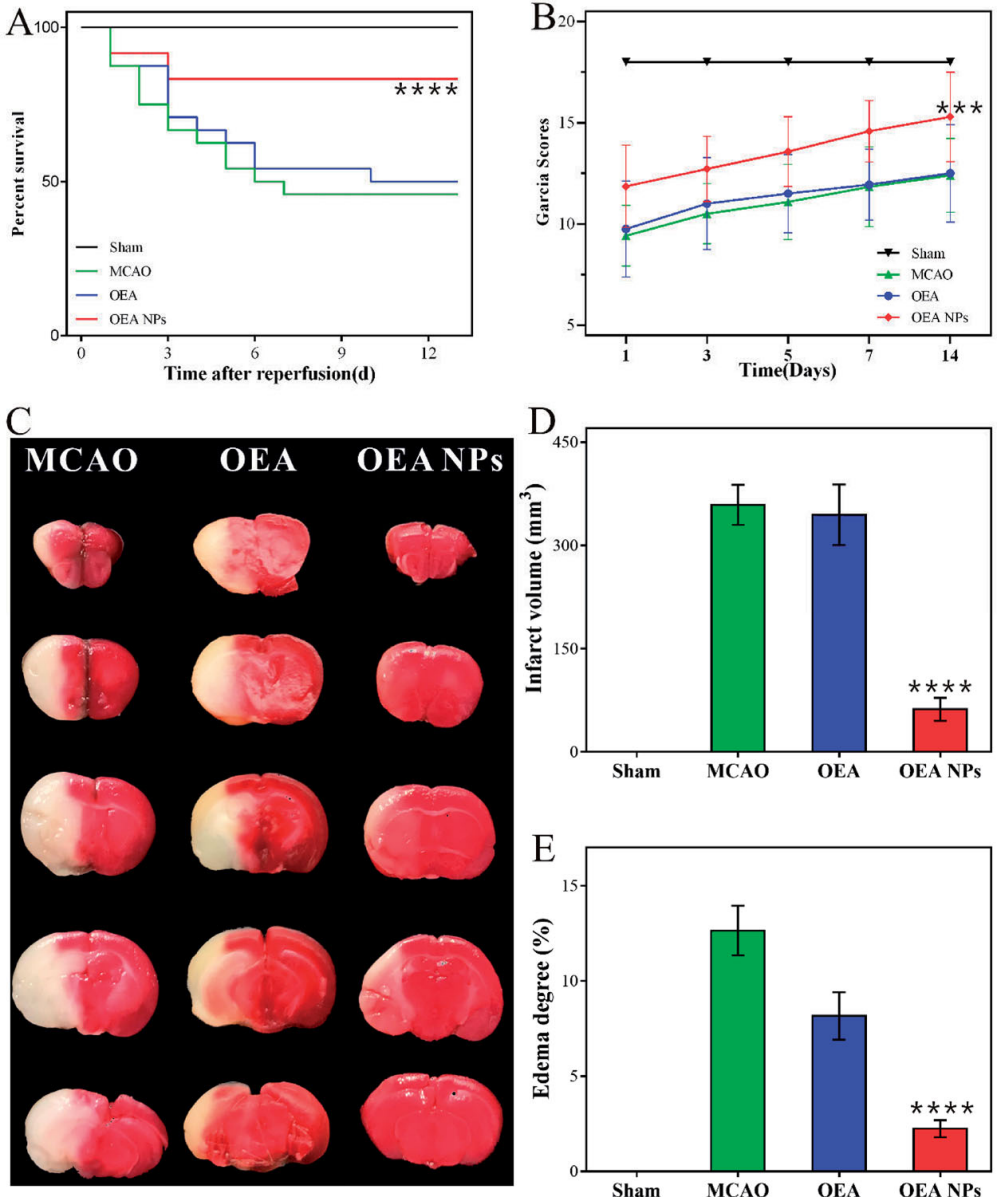
Therapeutic effects of the indicated formulations (0.9% NaCl (Sham), 0.9% NaCl (MCAO), OEA, and OEA NPs, [OEA] = 1.5 mg/kg) on cerebral damage after MCAO in rats. The survival rate (A) and the Garcia scores (B) of the rats administrated with the indicated formulations. The photos of the TTC-stained brain slices from the MCAO rats (24 h post-reperfusion) administrated with the indicated formulations (C) and the statistical data of the cerebral infarct volume (D), and the cerebral edema (E). ***p < .001, ^****^p < .0001, compared to MCAO group).

**Figure 3.**
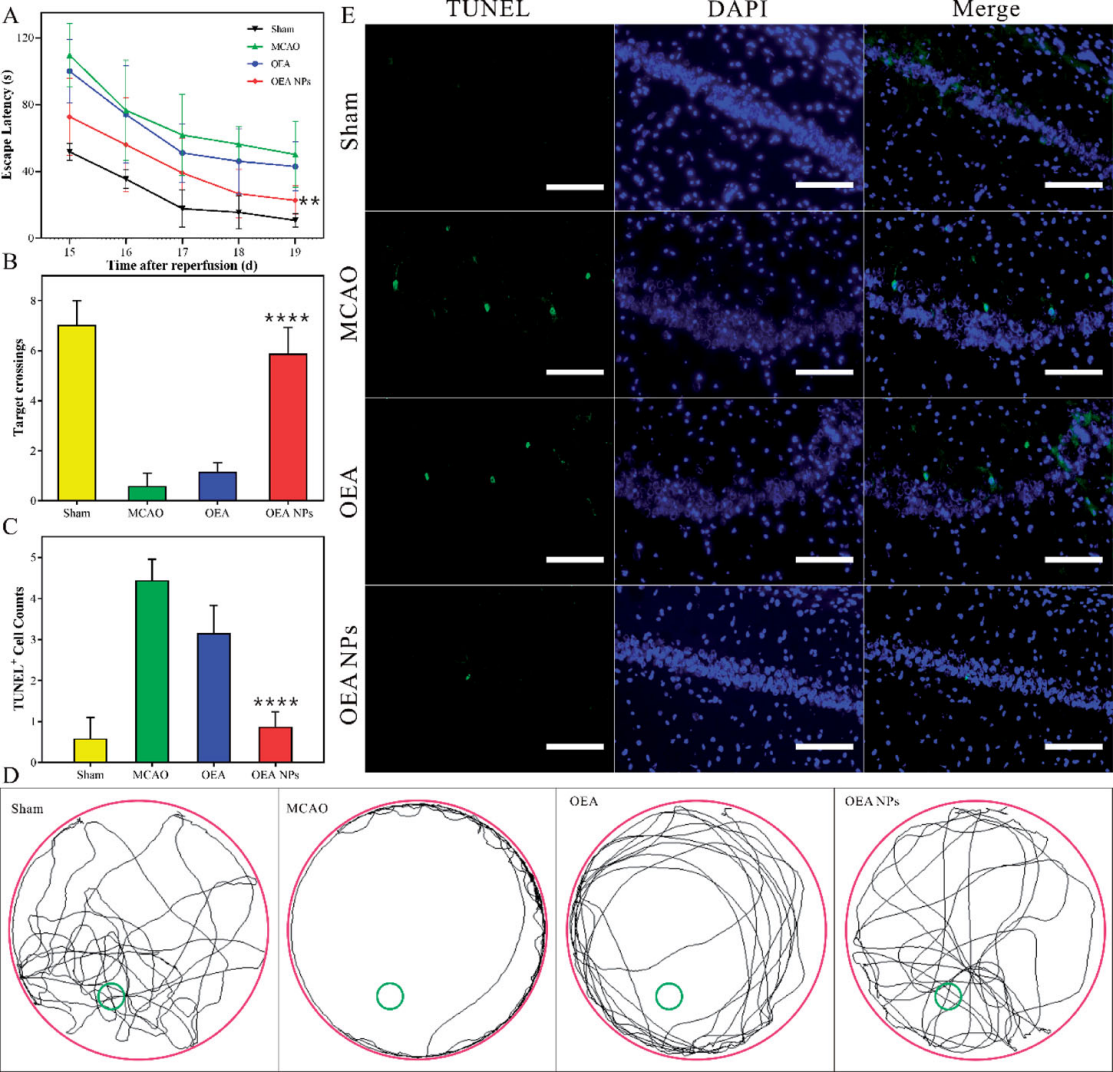
The evaluation of the behavior ability and the apoptosis of neurons. The effect of the formulations on MCAO-induced spatial cognitive deficits. The escape latency (A), the number of target platform crossings (B), and the swimming traces (D) in the hidden platform trails. Representative pictures (E) and the statistical data (C) of the hippocampal CA1 stained with TUNEL (scale bars: 125 µm). Data are expressed as mean ± SEM. *n* = 10–12 rats per group. ^****^p < .0001, compared to MCAO group).

### In vitro drug release study

In the oral OEA NDDS (OEA-SPC NPs), the OEA-SPC was self-assembled without other phospholipids, and OEA was distributed in the lipid bilayer. Differ from OEA-SPC NPs, OEA NPs integrated the drug in the interior layer of the liposomes. As to the liposomes based on hydrogen bonds, the sustained drug release came from the lipid-bilayer architecture and the hydrogen bonds between the drug and lipid. With a regular and tight structure, the lipid-bilayer architecture could restrict the release of the drug. And the drug in the outer layer of the liposomes should release faster than those in the interior layer, theoretically. Hence, we think that the OEA NPs should possess better-sustained drug release than that of OEA-SPC NPs. However, it could be seen from Figure S4 that two kinds of liposomes exhibited exactly similar drug release profiles. Both liposomes exhibited a slight burst release within 4 h and a remarkably sustained, prolonged release in the next 32 h. Therefore, it might be the hydrogen bonds between OEA and SPC play a paramount role in the sustained release behavior. The results indicated that OEA would release slowly and stably in the blood circulation *in vivo*, mainly attributed to the formation of the OEA − SPC complex.

**Figure 4.**
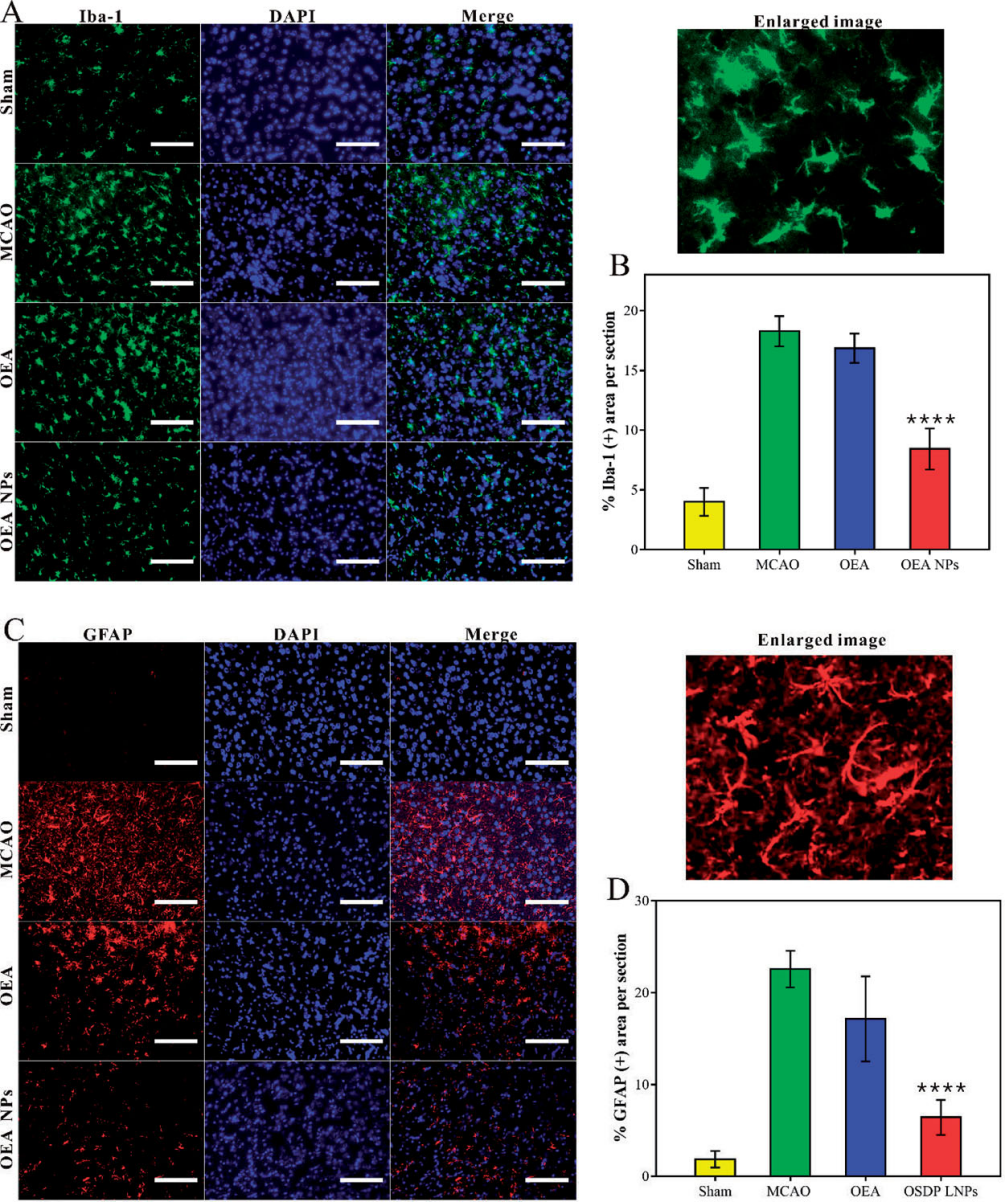
Quantification of the area of IBA-1 positive stain (A,B) and the area of GFAP positive stain (C,D) from the cortex around the ischemic focus. Scale bars: 125 µm. (^****^p < .0001, compared to MCAO group).

### The survival rate

With high mortality, stroke has been a leading cause of death worldwide, and reducing death rates must be the top priority to a formulation for stroke. So the survival rate of the MCAO rats administrated with the indicated formulations (0.9% NaCl [MCAO], OEA and OEA NPs, ([OEA] = 1.5 mg/kg)) were recorded. As shown in Figure 2(A), only 45.8% of the MCAO rats could survive for 14 d, more than half MCAO rats died without treatment. With the administration of free OEA, the survival rate was a little bit improved to 50%, which had no significant difference from that of the MCAO group. In comparison, *via* the administration of OEA NPs, only a fraction of the MCAO rats died from cerebral ischemia, and the survival rate was significantly increased to 83.3%. The result indicated that the administration of OEA NPs might build up resistance to cerebral ischemia and extend the time window for beneficial reperfusion.

### The behavioral assessment

Since the patients’ behavior was one of the most important and intuitionistic indicators in the clinic, the neurological functional deficit scores of the MCAO rats were assessed *via* Garcia method. The Garcia scores of the rats were evaluated at 1, 3, 5, 7, and 14 d after reperfusion. As shown in Figure 2(B), the sham-operated rats got full marks throughout the assessment, while the operated rats without treatment only got about 10 points in the 1st assessment and about 12 points in the last assessment, illustrating the irretrievable neurological function deficit of the MCAO rats. And the scores were almost the same as those of the rats administrated with OEA. The result indicated that OEA seemed to have no effect on improving the Garcia scores of the MCAO rats, mainly owing to its extremely low bioavailability. On the contrary, the rats administrated with OEA NPs got significantly higher scores than those of the other two operation groups (*p* < .01). The results illustrated that OEA could obviously mitigate and ameliorate the neurological function deficit caused by cerebral ischemia and the subsequent reperfusion.

### The cerebral infarct volume and the cerebral edema

The cerebral infarct volume and cerebral edema are another two important indicators, which could intuitively reflect the condition of stroke. As shown in Figure 2(C) and the statistical data (Figure 2(D–E)), nearly half of the brains from the MCAO rats without treatment were infarcted (358.8 ± 29.14 mm^3^) and the cerebral edema degree was as high as 12.64 ± 1.31%. Both data stated that these rats suffered from severe brain damage after operation. And the MCAO rats seem to get no significant progress from the treatment of OEA. Their infarct volume was 344.5 ± 44.0 mm^3^ and the cerebral edema degree was 8.16 ± 2.16%. These data had no significant difference from those of the MCAO group, which was in accordance with our previous study (Yang et al., 2019). In comparison, the rats administrated with OEA NPs possessed much slighter brain damage compared with the other operated rats. The infarct volume was reduced to 61.79 ± 16.75 mm^3^ and the cerebral edema degree was reduced to 2.24 ± 0.79%. With the same cerebral ischemia time, the administration of OEA NPs could build up resistance to the cerebral ischemia and hence, reduce brain damage. The mechanism was speculated to be that the acute ischemic brain tissues could be preserved as penumbral tissues, which would bounce back with timely reperfusion.

### The Morris water maze task

As an important computational function, learning and memory are usually impaired by ischemia reperfusion. To access the effect of OEA NPs on spatial learning and memory, the water maze task was employed between 15 and 20 d after reperfusion. The escape latency was recorded in 5-d training (Figure 3(A)). The sham-operated rats used the minimum time to find the platform, illustrating their superior learning and memory ability. Since the operation had cut off the blood supply of the brain for 90 min, the MCAO rats possessed inreversible and serious neurologic deficits. It took more time for the MCAO rats to find the platform. And escape latency of the MCAO rats treated with OEA had no significant difference from that of the MCAO rats, suggesting that the deficit could not be improved *via* the administration of OEA. On the contrary, the OEA NPs seemed to play a significant role in protecting the rats against the operation. As a result, the MCAO rats with the treatment of the OEA NPs used much less time to find the platform than the other operated rats. After 5 d of training, the platform was removed and the rats were exposed to the water maze again. The times they crossed the removed platform and their traces were recorded. As shown in Figure 3(B), the MCAO rats without treatment seemed not to remember the position of the removed platform and crossed the position once at most. The result illustrated the gravely impaired memory, which could not be repaired *via* the administration of OEA. However, with the treatment of OEA NPs, the MCAO rats did remember the removed platform, and they crossed the position about 5 times. Their performance had no significant difference from that of the sham-operated rats. The same conclusion could be drawn from the traces of the rats. If the rats did not remember the removed platform, they just swam around without specific direction. Otherwise, they would always swim around the position of the removed platform. As shown in Figure 3(D), the rats of the nanodrug group and sham group always swam around the position of the removed platform, indicating their significant purpose and strong memory for the removed platform. Therefore, OEA NPs greatly ameliorated ischemia-induced spatial memory impairment.

### The apoptosis of neurons

The intractable sequelae of stroke mostly resulted from the apoptosis of neurons, which were really hard to recover. And the impaired memory and cognition might result from the apoptosis of neurons in hippocampal CA1. Herein, TUNEL staining was employed to evaluate the ischemia-induced apoptosis in hippocampal CA1. As shown in Figure 3(C,E), there was no apoptotic cell in hippocampal CA1 of the sham-operated rats, suggesting their normal brain state. And a significant increase in apoptotic cells was observed in rats of the MCAO group and OEA group. This result indicated that the ischemia reperfusion could induce the apoptosis of the neurons, which could not be prevented by the administration of free OEA. Nevertheless, the administration of OEA NPs could largely reduce the apoptosis of the neurons, which might relate to the ameliorative learning and memory ability. The result indicated the excellent neuroprotective effect of OEA NPs.

### The inflammation

In addition to the ischemia, the inflammation of reperfusion is another primary cause of the serious sequelae, which might induce severe brain damage. The brain inflammation was mostly mediated by microglia and astrocyte, which could be marked by Iba-1 and GFAP, respectively. Hence, the expressed Iba-1 and GFAP in the cortex were assessed to access the inflammation after reperfusion. As depicted in Figure 4, both markers indicated that the inflammation from the brain of the nanodrug group was as slight as those from the sham-operated rats. However, the other operated rats possessed a significantly increased inflammation. The results suggested that the inflammation of reperfusion was inhibited to a low level with the administration of OEA NPs, and therefore, OEA NPs could provide significant neuroprotective effects. This section may be divided into subheadings. It should provide a concise and precise description of the experimental results, their interpretation, as well as the experimental conclusions that can be drawn.

## Conclusions

In summary, the study demonstrates a new kind of OEA loaded liposomes for efficient stroke therapy. The hydrogen bonds between OEA and SPC totally changed the form of OEA and would allow for more effective drug loading and sustained drug release. Hence, the bioavailability of OEA was highly increased. With systemic *in vivo* experiments, the survival rate, the behavioral score, the spatial learning, and memory ability, the cerebral infarct volume, and the edema degree of the MCAO rats were greatly improved *via* the administration of OEA NPs. And the apoptosis of the neurons and the inflammation within the brain were significantly inhibited. These results forcefully indicated that the OEA NPs had potential applications in stroke therapy, and may open new opportunities for the clinic use of OEA.

## Ethics approval and consent to participate

All procedures were carried out in accordance with the National Institutes of Health Guide for the Care and Use of Laboratory Animals and were approved by the Animal Ethical and Welfare Committee of Central South University.

## Supplementary Material

Supplemental Material

## Data Availability

The authors confirm that the data supporting the findings of this study are available within the article and its supplementary materials.
