# Correction notice

**DOI:** 10.1080/10717544.2022.2109284

**Published:** 2022-08-08

**Article title:** N-oleoylethanolamine_phosphatidylcholine complex loaded, DSPE-PEG integrated liposomes for efficient stroke

**Authors:** Xiangrui Yang and Shichao Wu

**Journal:** *Drug Delivery*

**Bibliometrics:** Volume 28, Number 1, pages 2525–2533

**DOI:** https://doi.org/10.1080/10717544.2021.2008058

As per author’s suggestions the figure 3 and 4 are replaced in the published article.

