# Supplementary material for: N-oleoylethanolamine − phosphatidylcholine complex loaded, DSPE-PEG integrated liposomes for efficient stroke
Source: Drug Deliv. 2021 Nov 29;28(1):2525–33. doi: 10.1080/10717544.2021.2008058 (PMC8635618; doi:10.1080/10717544.2021.2008058)
Supplement: Supplemental Material [file IDRD_A_2008058_SM9919.docx]

Supporting Information

N-oleoylethanolamine - Phosphatidylcholine Complex Loaded, DSPE-PEG Integrated Liposomes for Efficient Stroke Therapy

Xiangrui Yang, and Shichao Wu*


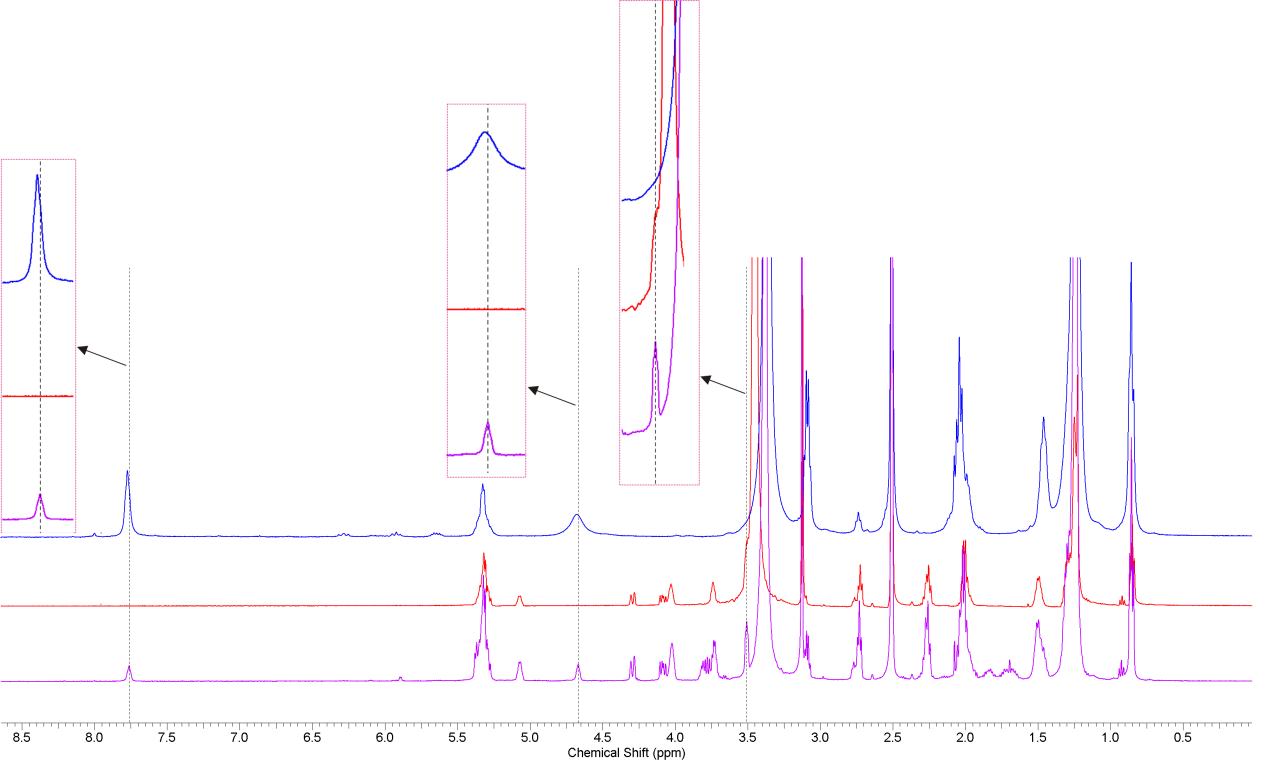


Figure S1: The H^1^NMR spectra of OEA, SPC, and OEA-SPC complex.





Figure S2. the TEM image of OEA NPs (OEA-SPC:DSPE-PEG=1:1.75).





Figure S3: The size distribution of OEA NPs.





Figure S4: The in vitro drug release of free OEA, OEA-SPC NPs, and OEA NPs.

Table S1. Factors in orthogonal-design experiments

| Factor | 1 | 2 | 3 |
| --- | --- | --- | --- |
| 1. The ratio of OEA-SPC to DSPE-PEG | 1:1 | 1:1.3 | 1:1.75 |
| 1. The ultrasonic power | 100W | 200W | 400W |
| 1. The concentration of the DSPE-PEG | 0.5 mg/mL | 0.75 mg/mL | 1 mg/mL |

Table S2. Results of orthogonal-design experiments

| Factors | Drug loading(wt%) | Size(d, nm) | Zeta potential(mV) | PDI |
| --- | --- | --- | --- | --- |
| A1,B1,C1 | 9.24±0.33 | 230.5±24.3 | - 13.4±1.2 | 0.123±0.012 |
| A1,B2,C1 | 9.31±0.16 | 121.2±13.1 | - 13.9±1.4 | 0.131±0.018 |
| A1,B3,C1 | 8.95±0.36 | 52.4±7.6 | - 8.6±2.3 | 0.216±0.035 |
| A2,B2,C1 | 8.21±0.18 | 76.3±5.6 | - 15.4±3.1 | 0.104±0.021 |
| A3,B2,C1 | 7.26±0.15 | 55.1±2.6 | -13.3±4.7 | 0.086±0.011 |
| A2,B2,C2 | 8.35±0.23 | 65.6±3.8 | - 14.7±4.8 | 0.185±0.032 |
| A2,B2,C3 | 8.38±0.27 | 56.3±4.4 | - 15.9±4.2 | 0.205±0.045 |
